# New measures against chronic kidney diseases in Japan since 2018

**DOI:** 10.1007/s10157-019-01786-7

**Published:** 2019-09-09

**Authors:** Akira Fukui, Takashi Yokoo, Masaomi Nangaku, Naoki Kashihara

**Affiliations:** 1 Division of Nephrology and Hypertension, Department of Internal Medicine, Jikei University School of Medicine, Tokyo, Japan; 2 Division of Nephrology and Endocrinology, The University of Tokyo Hospital, Tokyo, Japan; 3 Department of Nephrology and Hypertension, Kawasaki Medical School, Okayama, Japan

**Keywords:** Chronic kidney disease (CKD), Criteria for referral, Key performance indicators (KPIs), Ministry of health, Labor and welfare

## Abstract

Since 2008, the Japanese government has started measures against chronic kidney disease (CKD), and steady changes have been achieved, including a decrease in the age-adjusted rate of new dialysis introduction. However, the total number of dialysis patients has not declined because of the progression of aging. Therefore, the Japanese government has started more concrete measures since 2018. It aims to prevent CKD exacerbation mainly by early referrals to nephrologists using “criteria for referral from a primary care physician to a kidney specialist/specialized medical institution”. In addition, key performance indicators are set to reduce the number of new dialysis patients from 39,000 in 2016 to ≤ 35,000 by 2028. We hope that you can refer to this measure all over the world and proceed with CKD measures. This report has been originally notified from the Ministry of Health, Labor and Welfare in Japanese. This is the English version of it.

## Introduction

The number of patients with kidney disease in Japan is increasing each year, and as of the end of 2006, ~ 260,000 patients were receiving dialysis therapy, indicating that the disease has a significant impact on public health. However, compared with preventive measures for lifestyle-related diseases and kidney failure, such as dialysis and transplantation, prevention of chronic kidney disease (CKD) exacerbation has not been clarified as a target of these measures. Therefore, since October 2007, “The Kidney Disease Control Commission Meeting” examined the ideal measures against kidney diseases in Japan. The results were summarized as “Ideal kidney disease measures in the future”, which indicate the future direction of measures against kidney disease with the following goals: “to prevent aggravation of renal function abnormality and to arrest progression that leads to the introduction of dialysis due to chronic renal failure”, and “to suppress the development of cardiovascular diseases, such as cerebrovascular disease and myocardial infarction, associated with CKD”.

Through the measures implemented in the subsequent 10 years, steady changes were achieved, including a decrease in the age-adjusted rate of new dialysis introduction; however, the number of patients receiving dialysis has not yet shown a decreasing trend. The number of patients with kidney disease resulting from lifestyle-related diseases is expected to continue to increase in the future as the population ages.

Therefore, the first “Kidney Disease Control Commission Meeting” was held in December 2017 to promote measures against kidney disease and examine the future direction of measures against kidney disease over the course of four meetings. This commission aims to prevent CKD exacerbation through early detection and diagnosis of CKD based on few subjective symptoms, to implement and continue appropriate high-quality treatment, and to maintain and improve the quality of life (QOL) of patients with CKD, including those receiving dialysis and kidney transplant. The activities identified for future implementation are organized into each of the following five categories: “raising public awareness”, “improving regional health care provisions”, “improving the level of medical care”, “developing human resources”, and “promoting research and development”. In addition, key performance indicators (KPIs) are set to reduce the number of new dialysis patients to ≤ 35,000 by 2028. The contents of these discussions at the “Kidney Disease Control Commission Meeting” are summarized as a meeting report described herein.

This report is expected to contribute to the wider recognition of the importance of measures against kidney disease. Moreover, the public awareness of CKD is anticipated to improve among patients and their families, healthcare workers, and government agencies such that the measures against kidney disease are implemented.

## Current status of kidney diseases

- Characteristics of kidney disease
  Omitted due to space limitations.
- Causes of kidney disease
  Omitted due to space limitations.
- CKD
  Omitted due to space limitations.
- Epidemiology of CKD
  The number of patients with CKD is ~ 13 million, which equates to approximately one in eight adults, indicating its high prevalence (2012 CKD clinical practice guidelines). In recent years, the rate of increase in the number of patients on dialysis has reduced, but it is not decreasing and reached 329,609 by the end of 2016. In addition, the number of new patients receiving dialysis has been stable in recent years, with the number in 2016 being 39,344. As renal function declines with age, aging of the population is likely to be a factor that increases the number of patients with CKD; however, the recent trend of the number of new patients receiving dialysis has not increased. This is considered to reflect the steady results of the measures against kidney disease implemented thus far. In addition, the average age of patients on dialysis in 2016 was 69.4 years (the current status of dialysis therapy in Japan, illustrated, the Japanese Society for Dialysis Therapy). In a report considering the aging of patients on dialysis, following adjustments for age to eliminate the influence of aging on the number of new patients receiving dialysis, a decrease in the rate of patients newly introduced to dialysis was observed compared with the number in 2008 (Wakasugi et al., The Japanese Journal of Nephrology, Volume 60, Issue 1).
  The most likely disease leading to dialysis included diabetic nephropathy, chronic glomerulonephritis, nephrosclerosis, and polycystic kidney in that order. Diabetic nephropathy has been the leading cause since 1998 and has accounted for ~ 43% with a flat trend. In contrast, nephrosclerosis caused mainly by hypertension or aging is increasing each year.
- Association between CKD and cardiovascular disease
  Patients with CKD are known to be at a high risk of cardiovascular diseases, including myocardial infarction and cerebral infarction. Renal failure is the seventh leading cause of mortality in Japan, following malignant neoplasms, heart disease, pneumonia, cerebrovascular disease, senility, and accidental injuries (2015 Vital Statistics Survey). Complications including heart disease and cerebrovascular disease, which are leading causes of mortality, are often the causes of mortality in patients with CKD, including those on dialysis. Therefore, CKD should be widely recognized as a serious life-threatening disease with a substantial impact on public health, and appropriate measures should be taken.
- Measures against CKD irrespective of primary disease
  Among the measures against kidney disease, the importance of those against diabetic nephropathy is widely recognized. For instance, the Diabetic Nephropathy Aggravation Prevention Program was established in 2016. However, there are measures against CKD irrespective of the primary disease; treatment principles, including blood pressure and blood glucose control, and advice to reduce sodium intake are common. The implementation of measures against lifestyle-related diseases including nephrosclerosis, absolute numbers of which remain small but are increasing each year, and CKD, such as the control of intractable diseases (chronic glomerulus nephritis) that are prevalent in young patients and patients on long-term dialysis, is expected to lead to more effective and efficient measures.

## Further advancement of measures against kidney disease (summarized in Fig. 1)

1. Overall goals of measures
  To prevent CKD exacerbation while maintaining and improving the QOL of patients with CKD, including those receiving dialysis and transplantation, through early detection and diagnosis of CKD based on few subjective symptoms and implementation and maintenance of appropriate high-quality treatment at an early stage.
  To achieve the above goals, the government and related academic societies will conduct progress management of measures based on this report using methods such as the evaluation indices. It is desirable that the status be published, for example, on their website. In addition, in cases where achieving KPIs is difficult during management process, the government and related academic societies will examine and work on the measures while reviewing the approaches to be performed and working on achieving the goals.

**Fig. 1:**
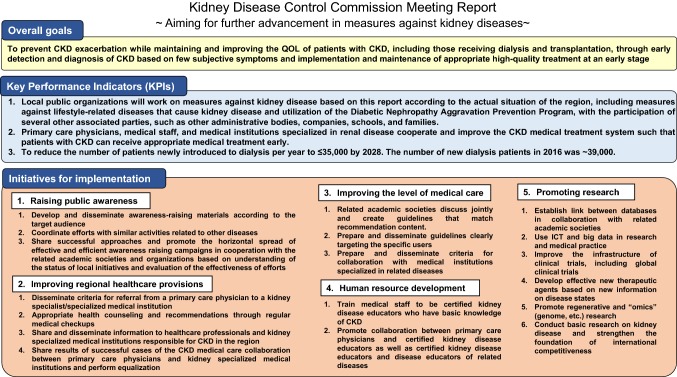
Overview of measures against kidney disease

*KPIs to be achieved and evaluation indices*

- Local public organizations will work on measures against kidney disease based on this report according to the actual situation of the region, including measures against lifestyle-related diseases that cause kidney disease and utilization of the Diabetic Nephropathy Aggravation Prevention Program, with the participation of several other associated parties, such as other administrative bodies, companies, schools, and families.
  (Evaluation indices)
  The situation of the approaches in each municipality
  The number of municipalities utilizing the diabetic nephropathy aggravation prevention program, etc.
- Primary care physicians, medical staff, and medical institutions specialized in renal disease cooperate and improve the CKD medical treatment system such that patients with CKD can receive appropriate medical treatment early.
  (Evaluation indices)
  Referral rate to a medical institution specialized in renal disease in accordance with the referral criteria.
  Reverse referral rate from a medical institution specialized in renal disease to a primary care physician.
  Number of healthcare workers, such as primary care physicians who treat patients with CKD in the area.
- To reduce the number of patients newly introduced to dialysis per year to ≤ 35,000 by 2028. The number of new dialysis patients in 2016 was ~ 39,000.
  (Evaluation indices)
  The number of prefectures that achieve ≥ 5% reduction in 5 years or ≥ 10% reduction in 10 years from the year 2016 in the number of patients newly introduced to dialysis, etc.
  It is considered useful to also evaluate the number of patients introduced to dialysis per population, with age adjustment, and according to the primary disease at that time.

(2) Individual measures
  Regarding individual measures, we aim to address the current issues and will present future approaches and evaluation indices for approaches to overcome these issues. This report examines and summarizes measures for each of the following five categories that include implemented measures based on the “ideal measures against kidney disease in the future” put together in 2008: “raising public awareness”, “improving regional healthcare provisions”, “improving the level of medical care”, “developing human resources”, and “promoting research and development”.

**Fig. 2:**
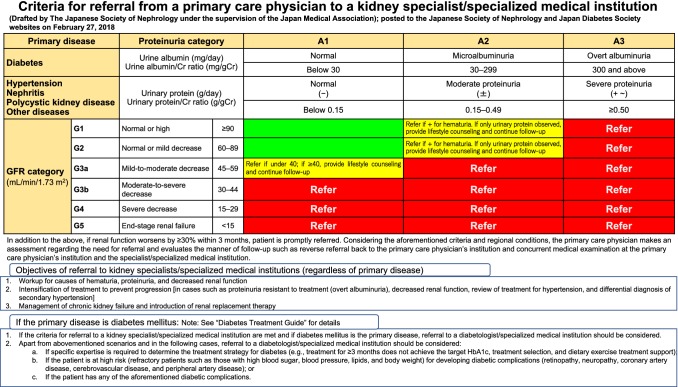
Criteria for referral from a primary care physician to a kidney specialist/specialized medical institution
  (iv) Evaluation indices
    Referral rate to a medical institution specialized in renal disease in accordance with the referral criteria.
    Reverse referral rate from a medical institution specialized in renal disease to a primary care physician.
    Number of healthcare workers, including primary care physicians who treat CKD, in the region, and so on.
③ Improving the level of medical care
  (i) Aims
    All medical workers involved in CKD treatment practice high-quality and appropriate medical treatment recommended by various guidelines and CKD exacerbation is prevented through improving cooperation with related disease treatment.
  (ii) Issues
    Various guidelines are named inconsistently, and it is unclear who should use them.
    Aspects of recommended content are inconsistent among various guidelines.
    Various guidelines are not sufficiently disseminated to medical staff and primary care physicians.
    Aspects of collaboration criteria between medical institutions specialized in renal disease and those specialized in related diseases, such as diabetes, are unclear.
  (iii) Approaches to be implemented in the future
    Related academic societies will discuss jointly and create guidelines that match recommendation content.
    Related academic societies will prepare guidelines clearly targeting the specific users, including patients, medical staff, and primary care physicians, and disseminate them in cooperation with related organizations and academic societies.
    Related academic societies will prepare and disseminate criteria for collaboration with medical institutions specialized in related diseases.
  (iv) Evaluation indices
    Creation of interdisciplinary guidelines.
    Dissemination rate of various guidelines in each target.
    Implementation rate of medical treatment recommended by various guidelines.
④ Developing human resources
  (i) Aims
    To increase the number of healthcare workers involved in CKD treatment to enrich the CKD medical care system. Although there is a shortage and uneven distribution of nephrology specialists, human resources need to be developed, such as nurses/public health nurses, registered dietitians, and pharmacists with a basic knowledge about CKD, who will also collaborate with doctors other than nephrology specialists. In regions where there are few medical institutions specializing in kidney disease, collaboration between treatment advisors and primary care physicians is expected to lead to improvements in the CKD medical care system.
  (ii) Issues
    There is a shortage and uneven local distribution of medical staff involved in CKD treatment.
    Collaboration between medical staff involved in CKD treatment and those involved in CKD-related diseases is insufficient.
  (iii) Approaches to be implemented in the future
    The related academic societies will train medical staff, including nurses/public health nurses, registered dietitians, and pharmacists, to be certified kidney disease educators who have basic knowledge of CKD.
    Related academic societies will promote collaboration between primary care physicians and certified kidney disease educators as well as between certified kidney disease educators and disease educators of related diseases.
  (iv) Evaluation indices
    The number of certified kidney disease educators in the region.
    The number of cases of collaboration between certified kidney disease educators and disease educators of related diseases, etc.
⑤ Promoting research and development
  (i) Direction of promoting research and development
    Research based on medium- to long-term research and development goals set by the government, such as the “research and development promotion plans in medical field”, should be promoted in an all-Japan system in which related academic societies and organizations, the government, local public organizations, and companies coordinate closely.
    The government should promote research that contributes to the achievement of the overall goals of this report.
    The government should cooperate with related academic societies to recommend and promote policies, implement measures based on this report, and promote research on progress management.
(ii) Research examples in the direction of research and development promotion
  Building links between databases by strengthening cooperation with related academic societies, in addition to cooperation on diabetic kidney disease (DKD) between the Japanese Society of Nephrology and the Japan Diabetes Society. Cooperation between related academic societies for cardiovascular disease, such as the Japanese Circulation Society, is also important.
  Utilization of information and communication technology as well as big data for research and clinical practice.
  Establishing the base of clinical trials, including joint international studies.
  Developing novel effective therapeutic agents based on the elucidation of pathological conditions.
  Promoting regenerative and omics (e.g., genomic) research.
  Strengthening basic research and global competitiveness on kidney disease.

① Raising public awareness
  (i) Aims
    To raise awareness of CKD among healthcare workers and government agencies as well as among patients and their families in Japan irrespective of age, to build a system through which more individuals can practice measures against kidney disease, and to further promote the measures against kidney disease through planned, efficient, and effective public awareness campaigns.
  (ii) Issues
    The following recognition and knowledge of CKD is not sufficiently disseminated: CKD is a life-threatening disease affecting many individuals; however, it is treatable, and early detection and treatment are important.
    The levels of increase in awareness according to the target audience, including physicians, medical staff, government agencies, patients with CKD, general public, the elderly, and children, are not sufficiently examined.
    The implementation status of activities to improve awareness is not fully understood, and the effects are not sufficiently evaluated or verified. Therefore, effective awareness raising campaigns are not undertaken.
    Successful examples are not shared among healthcare workers, related academic societies, and government agencies, and the horizontal spread of successful examples has not progressed sufficiently.
  (iii) Approaches for future implementation
    The government will cooperate with the related academic societies and examine and organize the contents that should be recognized and disseminated according to the target audience. Based on this, the materials for raising awareness will be developed and disseminated.
    The related academic societies will determine the person in charge, who plays a central role in the measures against kidney disease in the region, including raising awareness for each prefecture. Based on this, in cooperation with local public organizations centering on the person in charge, activities to raise awareness will be promoted. In addition, information relating to the activities will be collected, the implementation status of the region will be determined, and the effects of activities will be evaluated. In addition, dissemination activities coordinated with similar activities related to other diseases, including diabetes, hypertension, and cardiovascular diseases, are considered to be effective and efficient.
    The government and local public organizations will share successful approaches and promote the horizontal spread of effective and efficient awareness raising campaigns in cooperation with the related academic societies and organizations.
  (iv) Evaluation indices
    The number of activities performed to raise awareness in each prefecture.
    The number of citizen-open lectures and similar events conducted.
    Awareness levels of CKD, etc.
② Improving regional healthcare provisions
  (i) Aims
    The large number of patients with CKD presents a challenge when treatment is performed only at medical institutions specialized in renal disease. However, when symptoms are mild, treatment is mainly provided by general internal medicine practice, including blood pressure and blood glucose control, and advice to reduce salt intake. Once the disease advances, specialized medical treatment is required, including the selection and preparation of complex preventive measures and optimal renal replacement therapy (hemodialysis, peritoneal dialysis, and kidney transplantation). Therefore, the establishment of a medical care system that can detect and diagnose CKD early and initiate and continue appropriate treatment at an early stage is required. This will be enabled by promoting collaboration between primary care physicians and medical institutions specialized in renal disease through referral/reverse referral and a two-doctor system, with the cooperation of medical staff.
  (ii) Issues
    The criteria for referral from a primary care physician to medical institutions specialized in renal disease or in diabetes are not sufficiently recognized.
    In each region, there is insufficient knowledge of the medical institutions specialized in renal disease, with which primary care physicians should cooperate with.
    Reports of cases of successful cooperation between primary care physicians and medical institutions specialized in renal disease are not sufficiently shared among government agencies, related academic societies, and related organizations. Therefore, equalization of regional health care provisions has not progressed.
  (iii) Approaches to be implemented in the future
    Related academic societies and related organizations will cooperate with the government and local public organizations, and widely disseminate the following among those involved in CKD treatment: “criteria for referral from a primary care physician to a kidney specialist/specialized medical institution (Fig. 2)” and “criteria for referral from a primary care physician to a diabetes specialist/specialized medical institution, based primarily on diabetes treatment guidelines”.
    Health check-ups provide a good opportunity to detect CKD or risk factors for CKD, such as diabetes and hypertension. Therefore, each institution conducting health check-ups will provide health guidance and consultation recommendations referring to “examples of health checkup judgment and counterplans for CKD”.
    The related academic societies and organizations will cooperate with the government and local public organizations, share information with healthcare workers, including primary care physicians who are treating CKD, in the region and medical institutions specialized in renal disease, enabling collaboration. In addition, the person in charge, determined by related academic societies, who plays a central role in implementing measures against kidney disease in the region, is expected to actively participate in adjustment among local public organizations and related organizations.
    Government agencies, related academic societies, and organizations will share results of successful cases of the CKD medical care collaboration system and perform equalization.

## Overview of measures against kidney disease

**Fig. 3:**
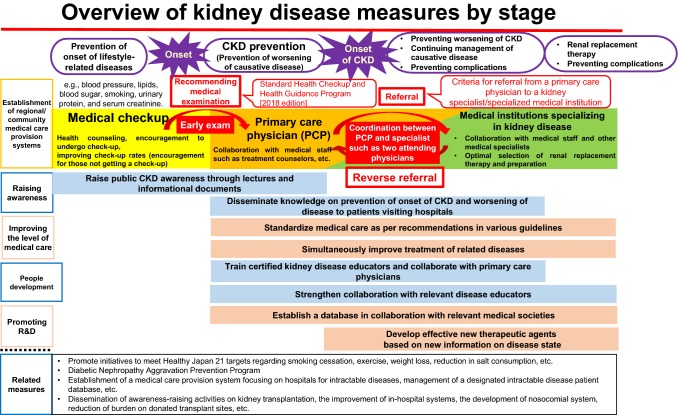
Overview of kidney disease measures by stage
  Among the KPIs for the overall goals, the goal “to decrease the number of patients newly introduced to dialysis to ≤ 35,000” reflects the sum of all measures, including the related measures. The most important measures for achieving this KPI are the prevention of CKD exacerbation by establishing a CKD medical treatment system, which enables appropriate recommendations to visit a primary care physician based on health examination results and referral from a primary care physician to a nephrology specialist in accordance with referral criteria. A successful example of this approach is the case of Kumamoto City, where a reduction of ~ 18% (from 295 to 243) was achieved in the number of dialysis patients introduced to new dialysis between 2009 and 2016. Improvements in health examination rates and prevention of the onset and exacerbation of lifestyle-related diseases are also measures that can reduce the number of new dialysis patients and should thus be promoted. However, diabetic nephropathy often develops > 10 years after the onset of diabetes, and it takes a long time for the results of these measures to appear as a decrease in new dialysis patients. Therefore, efforts from a long-term perspective are necessary.
  Furthermore, to achieve improvements in the QOL of patients with CKD, in addition to preventing CKD exacerbation, it is important to prevent various complications; to select and prepare optimal renal replacement therapy (hemodialysis, peritoneal dialysis, and kidney transplantation) for each patient; and to support patients to balance treatment and work.
② Efforts related to kidney disease by the Ministry of Health, Labor and Welfare
  To gain a wider perspective of measures against kidney disease, efforts of the Ministry of Health, Labor and Welfare related to kidney disease have been introduced. To further promote measures against kidney disease, it is important for the concerned parties to closely cooperate and implement measures with a broad perspective based on a comprehensive understanding of the wider setting, including the related measures.
  (i) Measures against lifestyle-related disease
    Lifestyle-related diseases, such as diabetes, hypertension, and dyslipidemia, are risk factors for CKD, and a reduction in the number of patients developing CKD can be expected following improvements in lifestyle habits, such as smoking cessation. Therefore, prevention of the onset and exacerbation of these lifestyle-related diseases and improvement of lifestyle habits hold a prominent position in measures against kidney disease.
    Omitted below due to space limitations.
  (ii) Measures against intractable diseases
    Act on Medical Care for Patients with Intractable/Rare Diseases (2014 law no. 50) was enforced on January 1, 2015, and measures including research promotion and improvements in living environment with medical treatment were implemented. As of April 2018, 311 diseases were designated as intractable diseases, which are the target diseases of medical subsidies. Among these, 19 diseases were related to the Japanese Society of Nephrology. The use of a database of patients with designated intractable diseases, in which the data described on the individual clinical survey are registered, was initiated in fiscal year 2017, and its utilization for research is expected. Furthermore, in fiscal year 2018, a new medical care delivery system was established, which centers on the intractable disease medical care cooperation hub hospitals of each prefecture, to enable early diagnosis and appropriate treatment that can be provided at more familiar medical institutions once diagnosed. The collaboration between a primary care physician and a medical institution specialized in renal disease, which is improved through medical treatment of intractable disease, is expected to be utilized in CKD treatment caused by factors other than intractable diseases.
  (iii) Transplantation medicine
    Omitted due to space limitations.

① Overview of measures against kidney disease according to the disease stage (Fig. 3)

## Conclusion

In the first to fourth Kidney Disease Control Commission Meetings, based on the “ideal measures against kidney disease in the future” compiled in 2008, the evaluation of goal achievement by measures in the last 10 years, the current status of kidney disease, and the specific methods to further promote measures against kidney disease were discussed. As the number of patients with CKD is expected to increase with the aging of population, further promotion of measures against kidney disease is required. By “visualizing” the progress and results of measures against kidney disease, in addition to sharing successful results, appropriately improving measures as necessary and equalizing continuous and effective measures against kidney disease are expected. In addition, as stated in Sect. 3, “for further advancement of measures against kidney disease”, among KPIs of overall goals, to achieve a “decrease in the number of new dialysis patients to ≤ 35,000 by 2028”, a model is first required for measures against kidney disease in the region based on this report, following which efforts are to be implemented to unify the model across the nation.

More efficient and effective promotion of measures against CKD can be expected by linking preceding measures against diabetic nephropathy, such as a program for preventing the exacerbation of diabetic nephropathy and CKD. For example, in clinical practice, medical workers are commonly involved in cases of diabetes and CKD, and there is a possibility that a network that has already been created can be utilized. In many governmental organizations, the involvement of various departments is necessary to implement measures against CKD. However, it is important that the person in charge of CKD measures is introduced to a local public organization as a liaison of collaboration with medical workers, such as a local person in charge designated by related academic societies.

In this report, measures against kidney disease have been examined and the future promotion of measures has been summarized; however, among the five categories, particularly aspects regarding improving public awareness and improving levels of medical treatment, are not unique to kidney disease, and it is expected that these categories will be utilized for measures against other diseases.

